# City Milk

**Published:** 1857-10

**Authors:** 


### CITY MILK.

A Merchant took his family seventy miles from the city for the purpose of securing for an infant child the advantages of a pure milk diet, and was at great pains to obtain his supplies from a thrifty farmer. In a few weeks, the entire scalp of the child became an ugly scab. On personal and minute inquiry, he ascertained that the milk he had been using was supplied by a cow fed on the swill of a distillery; on changing the milk, the disgusting scab disappeared, and the general health of the child became good, and by no other means than a change of milk.

"Milk from John Smith's Dairy," or some other man's, meets the eye in large letters, on freshly-painted milk-wagons, and on attractive sign-boards, about New York and Brooklyn; and the minds of the unsophisticated pass from the tidy-looking cart and glistening tin-cans to the cool "*spring-house*" of other years, where pans of luscious milk, covered with the thick yellow cream, set about on the cold, damp, and clean stone floor; and, for a moment, we feel young again!

But, take the Third Avenue cars or the Fulton Ferry-boat, and visit one of those "*dairies*" on Long Island or Manhattan. In long, low sheds, divided into stalls three feet wide and seven feet long, cows, from scores to hundreds, are standing, tied by the head, and stay there till they die, which averages six or eight months; or, they are taken out a few days before death, butchered, and sent to feed the unfortunate poor of New York. But the keepers sometimes miscalculate; the cow dies a day earlier than expected—milked in the morning, and at night the dead carcass lies in the yard! an almost daily occurrence. Open that cow; she has died of consumption, and the lungs are a "putrid black mass." As cows are almost daily dying, fresh, healthy ones are put in to supply their places, but they would die in a short time from the disease engendered there; so to moderate it, as in small-pox, the first thing done to a healthy cow is to cut a gash in her several inches long, and introduce into it some of the yellow, putrid matter of those already there, then tie up the wound with a rag; sometimes the part to which this virulent matter is applied rots off, in consequence of its more rabidly poisonous nature. Each cow drinks about twenty-five gallons of the swill on which they subsist, every twenty-four hours, and yields from one to six gallons of milk, such as it is: this milk is carted to New York, and sold at six cents a quart; and the people living within five miles of our City Hall pay for such milk, every year, over three millions of dollars. This swill is the refuse flowing from distilleries, causing the cows' teeth to fall out in a short time, so that they cannot eat hay or any solid food, even if they could get it: their tails sometimes rot off, disease falls into their hoofs, which prevents their standing; and when once down, they are often unable to rise; and in this condition, wallowing in their filth, they are continued to be milked until life is extinct! These facts, with others as revolting, were elicited in a trial before one of our city courts. Two-thirds of all the milk consumed in New York city is the yield of cows fed on distillery swill—of cows living and dying in their rottenness in those long, low sheds, divided into stalls three feet by seven, standing, lying down, and sleeping in the atmosphere of their foul excretions—never taking one breath of pure fresh air from the moment of their entrance until the day of their death! Fed on milk from such a source, it is no wonder that three hundred children die in a week, in summer-time, in this city. After this, can any thinking man use milk from the carts without most uncomfortable misgivings? About a third of the milk used in New York is out of healthy cows, and is consumed with no other admixture than cold water.

A grand achievement would it be, if some philanthropic man would go a hundred or two miles in the country, purchase the milk from the farmers in the neighborhood, require it to be delivered to him, before it is cold, from the cows, and furnish it to us daily, as pure, and fresh, and sweet, and rich, as any milk on any farmer's table. This can be done, and is done now for us; and the arrangement is as admirable as it is scientific. Three teaspoonsful color a cup of tea to the whiteness of half a cupful of the common milk; and in the hottest days of summer, there is not an atom of sourness observed on the top as large as a pin's point. It makes the very best rice pudding without eggs. Dilute it with three times its amount of pure water, stir it well, and thick, luscious cream will soon cover its surface, without the least sediment of any description. If put in air-tight cans and kept cool, it will keep as perfectly sweet for months as it was at the first moment of its milking. It is done thus:

Within an hour or two of milking, it is put into a vessel without air, and rapidly evaporated, until a gallon is reduced to a quart; this is put into air-tight vessels, and sent to various parts of the world. One pint of this concentrated milk, mixed with two pints of water, equals three pints of cream. One quart, mixed with five quarts of water, gives six quarts of milk, as rich as the best milk from common milk-carts: thus allowing the buyer to dilute his own milk—a privilege hitherto most tyrannically withheld.

The *saving* is one quart in six. Persons interested can make the experiment for themselves, by purchasing some at one hundred and seventy-three Canal street, of Gail Borden, Jr. & Co.—the gentlemen whose prepared meats kept Dr. Kane and all his company from perishing in their Arctic travels. The moment milk leaves the body, it begins to die, like the blood. It is the living milk that gives the highest life to the young; hence nature has ordered that all sucklings should receive the milk warm from the parent's bosom, while it possesses *all* its life. The moment it leaves its natural fountain, it begins to part with its life—and with it, its highest virtues. But to place milk in a *statu quo* condition, immediately after milking, in a locality where there are no distilleries, is an achievement of the highest importance in an alimentary and physiological point of view. This, Mr. Borden does.

The CONDENSED MILK keeps perfectly sweet for several days in mid-summer, if placed in an open vessel, in an ice-chest, or very cool cellar; hence, supplies can be had for two days at a time, and thus give to New York Sabbaths of rest and quiet—rest to our citizens from the clattering of hundreds of carts and the unearthly yells of as many milkmen—rest to the jaded horses and rest to their drivers, with the inestimable advantage of a perfectly pure milk, costing no more than that generally used. Hence we consider its general introduction a humanity.

